# Spatial Variation Characteristics of Polycyclic Aromatic Hydrocarbons and Their Derivatives in Surface Water of Suzhou City: Occurrence, Sources, and Risk Assessment

**DOI:** 10.3390/toxics13050403

**Published:** 2025-05-16

**Authors:** Jinxu Fan, Zhangwei Jing, Feng Guo, Jing Jia, Yu Jiang, Xiaoyu Cai, Shuting Wang, Hu Zhao, Xianjing Song

**Affiliations:** 1 National Research Center for Geoanalysis, Key Laboratory of Eco-Geochemistry, Ministry of Natural Resources, Beijing 100037, China; jinxufan2012@163.com (J.F.); j18771022691@163.com (Z.J.); langzho@sina.com (J.J.); shuting0429@163.com (S.W.); 17852261947@163.com (H.Z.); songxianjing210@163.com (X.S.); 2 Guizhou Academy of Testing and Analysis, Guiyang 550002, China; 3 Suzhou Sub-Bureau of Jiangsu Provincial Hydrology, Water Resources Survey Bureau, Suzhou 215000, China; jiangyu0515@aliyun.com (Y.J.); myname_caixy@163.com (X.C.)

**Keywords:** polycyclic aromatic hydrocarbons, derivatives, principal component analysis, ecological risk, Suzhou surface water

## Abstract

Polycyclic aromatic hydrocarbons (PAHs) and their substituted derivatives (SPAHs) are persistent organic pollutants derived from incomplete combustion of fossil fuels and industrial processes. These compounds are of global concern due to their carcinogenicity and environmental persistence. This study provides the first comprehensive analysis of PAH and SPAH contamination in Suzhou’s rapidly urbanizing watersheds, integrating ultra-high-performance liquid chromatography and high-resolution mass spectrometry with multidimensional risk assessment to address critical gaps in understanding pollutant dynamics in urban aquatic systems. Key findings reveal that SPAHs were significantly more abundant than parent PAHs (mean ∑19 SPAHs = 107.43 ng/L vs. ∑8 PAHs = 48.05 ng/L), with hydroxylated derivatives accounting for 67.9% of the total SPAHs, indicating active environmental transformation processes. Source apportionment identified coal combustion and industrial emissions as the dominant contributors (58.2% of PAHs), directly linking contamination patterns to localized anthropogenic activities. Notably, industrial zones exhibited unexpected toxicity hotspots, where SPAH toxicity equivalents (e.g., 3-OH-BaP) surpassed parent PAHs 2–5-fold, demonstrating substituent-driven toxicity enhancement—a critical finding for regulatory prioritization. This study advances the field by uncovering SPAHs as emerging risks in urban waterways, challenging traditional PAH-centric monitoring frameworks, and providing a novel integration of analytical chemistry and spatial risk mapping to guide targeted pollution control (e.g., prioritizing industrial discharges and non-exhaust traffic emissions). Furthermore, it highlights the urgent need for updated toxicological databases to account for substituted PAH derivatives and advocates for the regulatory inclusion of SPAHs. These insights underscore the necessity of adapting environmental policies to address complex pollutant mixtures in rapidly developing regions, emphasizing the replicability of the proposed framework for urban watershed management.

## 1. Introduction

Polycyclic aromatic hydrocarbons (PAHs) and their substituted derivatives (SPAHs) are a class of organic compounds composed of two or more fused aromatic benzene rings, characterized by unique cyclic structures and stable chemical properties [1,2]. As persistent organic pollutants, they have garnered significant attention in environmental science due to their carcinogenic, teratogenic, and mutagenic effects [3]. PAHs primarily originate from incomplete combustion of fossil fuels and petroleum product leakage, including industrial emissions, vehicle exhaust, coal combustion, and waste incineration [4]. Owing to their low water solubility, high lipophilicity, and elevated octanol–water partition coefficients, PAHs tend to adsorb onto suspended particulate matter or sediments, widely distributing across atmospheric, soil, sediment, and aquatic environments [5]. Consequently, they enter human bodies via food chains and drinking water, posing potential risks to aquatic organisms and public health [6]. The environmental fate of PAHs involves complex migration and transformation processes, entering surface and groundwater systems through atmospheric transport, particulate deposition, and stormwater runoff [7]. Their distribution and mobility in surface water are further influenced by geological structures, hydrodynamic conditions, and soil adsorption, leading to pronounced spatiotemporal variability in contamination patterns [8]. Common SPAHs such as nitrated PAHs (NPAHs), hydroxylated PAHs (OHPAHs), and oxygenated PAHs (OPAHs) share sources with parent PAHs [8,9,10]. Compared to their parent compounds, certain SPAHs exhibit higher toxicity, enhanced polarity, greater environmental persistence, and bioavailability, rendering PAH/SPAH contamination an increasingly critical research focus [3,8,11,12].

In the source apportionment of PAHs and SPAHs, diverse methodologies have been developed for the precise identification and quantification of pollution origins [13]. Isomer ratio methods (e.g., Phe/Ant, Flu/Pyr) utilize concentration ratios of specific PAH congeners to preliminarily differentiate between natural versus anthropogenic sources [14]. However, these ratios are susceptible to environmental variables (e.g., temperature, pH) and contamination history, introducing interpretative uncertainties, while overlapping ratios across sources limit their accuracy [15]. Receptor models based on mass balance principles enable quantitative assessment of source contributions, providing scientific foundations for targeted control strategies [16]. Yet, model construction requires extensive emission and environmental concentration datasets, often challenging to acquire, and idealized assumptions may deviate from real-world conditions, compromising result reliability [17]. Compound-specific isotope analysis (CSIA) distinguishes PAH sources (e.g., petroleum spills vs. combustion products) by analyzing stable isotopic ratios (δ13C, δ2H), offering direct evidence for source identification, particularly in complex environments [18,19]. Nevertheless, CSIA faces limitations, including high analytical costs, stringent laboratory requirements, and potential isotopic fractionation effects, necessitating meticulous calibration [20]. Comparatively, multivariate statistical approaches such as principal component analysis (PCA) coupled with multiple linear regression demonstrate superior efficacy in source analysis of aquatic PAHs/SPAHs. As an unsupervised learning technique, PCA reduces data dimensionality to extract principal components, revealing intrinsic patterns and dominant trends in contamination datasets. Without requiring prior knowledge, PCA identifies source categories (e.g., industrial emissions, traffic exhausts, petroleum leakage) and quantifies their contributions via absolute principal component scores (APCS), establishing a robust basis for risk assessment and mitigation strategies [21].

Despite extensive research on PAH/SPAH occurrence, concentration levels, and spatial distribution in atmospheric and soil matrices, studies on surface water environments remain limited, particularly in rapidly urbanizing regions [22,23,24,25,26]. Suzhou, a pivotal city in eastern China’s Yangtze River Delta, is renowned for its historical heritage, economic vitality, and unique geographical setting [27]. As a core urban hub and ecological priority zone, Suzhou’s surface water resources are vital to municipal water supply systems, directly impacting residents’ livelihoods and sustainable development [28]. However, accelerated industrialization and urbanization have imposed unprecedented environmental pressures, with surface water security threatened by multi-source pollution from industrial discharges, domestic sewage, and agricultural runoff [29,30,31,32]. Current research on PAHs and emerging derivatives in Suzhou’s surface waters remains scarce at regional scales, lacking systematic characterization and risk assessment [33]. This study pioneered a regional-scale investigation into the occurrence and spatial distribution of PAHs/SPAHs in Suzhou’s surface waters, employing multi-source apportionment models to decipher contamination origins and integrating ecological health risk analysis to quantify multi-media exposure risks. The findings aim to safeguard drinking water security, support sustainable urban development, and inform evidence-based pollution control policies.

## 2. Materials and Methods

### 2.1. Study Area and Sample Collection

**Figure 1:**
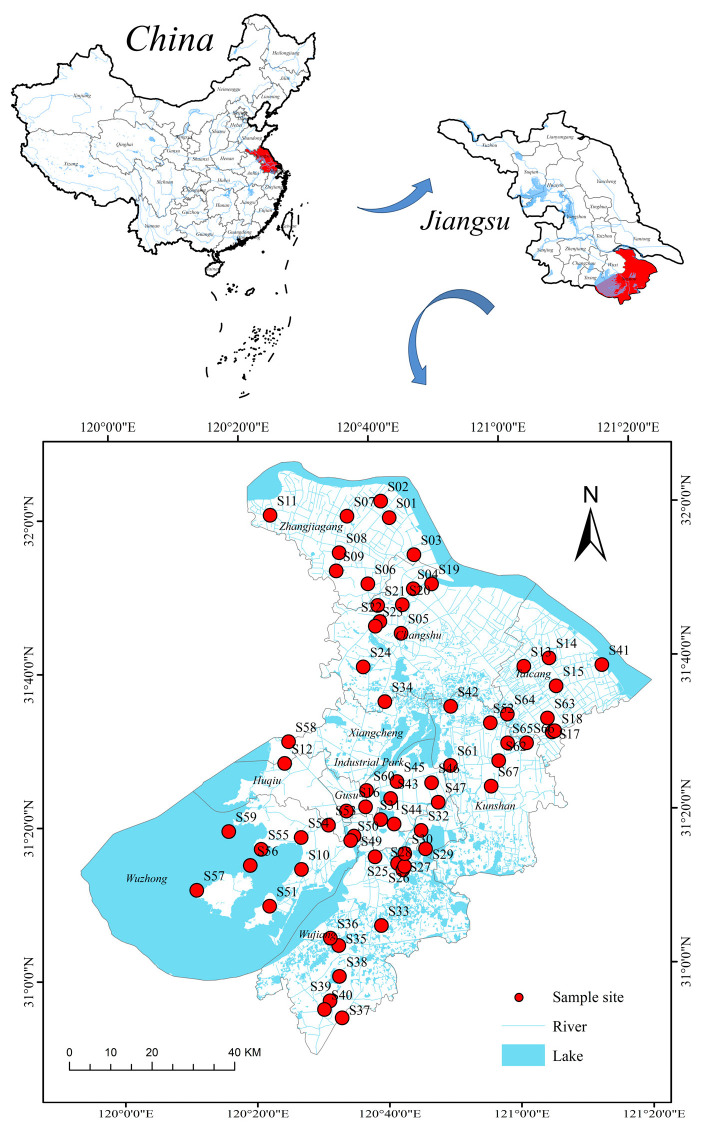
67 sampling points located in Suzhou, China.

Suzhou City is situated in the eastern Taihu Plain of the Yangtze River Delta, characterized by a subtropical monsoon marine climate with distinct seasons, abundant rainfall (annual average: 1100 mm), and a mean annual temperature of 15.7 °C [34]. Based on field surveys, 67 representative surface water samples were collected from rivers and lakes across Suzhou (sampling sites shown in Figure 1). Fifty mL of each water sample was stored in a precleaned centrifuge tube (washed three times with methanol and ultrapure water to avoid background contamination), and information such as sampling time, location, pH, and environmental conditions was recorded. The samples were stored in the dark at 4 °C and quickly transported to the laboratory for analysis.

### 2.2. Reagents and Instruments

Fifteen PAHs and 19 SPAHs (names and abbreviations are provided in Table 1) were analyzed. PAH standards (100 mg/L) and SPAH standards (100 µg/mL) were purchased from AccuStandard Inc. (AccuStandard Inc., New Haven, CT, USA). HPLC-grade acetonitrile and methanol were supplied by Merck KGaA (Merck KGaA, Darmstadt, Germany). Ultrapure water was prepared using a Milli-Q Advantage-10/Elix system (MilliporeSigma, Burlington, MA, USA). The key instruments included: an ST16R high-speed centrifuge (Thermo Fisher Scientific, Waltham, MA, USA), a Vortex-Genie 2 oscillator (Scientific Industries, Bohemia, NY, USA), an UltiMate™ 3000 online SPE–UHPLC system (Thermo Fisher Scientific) with a DGP 3600M dual-gradient pump, a TCC-3200 column oven, and a WPS-3000TSL autosampler, and a Q Executive Plus quadrupole-orbitrap high-resolution mass spectrometer (Thermo Fisher Scientific).

### 2.3. Pretreatment and Instrumental Conditions

#### 2.3.1. Water Sample Pretreatment

The samples were filtered through 0.22 μm membranes to remove suspended solids. Turbid samples were centrifuged at 5000 rpm for 10 min prior to filtration. For PAH analysis, 8 mL of filtered water was mixed with 2 mL of acetonitrile; for SPAHs, 10 mL of filtered water was directly transferred to vials.

#### 2.3.2. Instrumental Parameters

PAH analysis was conducted as described below. Online SPE steps included loading, washing, column regeneration, elution, and separation (the elution procedure is shown in Table A1) [35]. A 7.5 mL aliquot was loaded onto a Turboflow C18-XL SPE column (50 × 0.5 mm) and eluted onto an Agilent RRHD Eclipse Plus PAH column (1.8 µm, 3 × 100 mm) at 25 °C. Fluorescence detection (FLD-3000 RS) was used for quantification. SPAH analysis: similar chromatographic conditions were applied, except for the mobile phase (0.1% formic acid) and column temperature (30 °C). HRMS parameters: capillary temperature, 300 °C; corona discharge, 4.0 µA (positive), 10.0 µA (negative); full MS/dd-MS^2^ scan, *m*/*z* 100–900. Resolutions: 70,000 FWHM (MS1), 17,500 FWHM (MS2).

### 2.4. Quality Assurance and Control

Calibration curves (1–200 ng/L, R^2^ ≥ 0.99) were established. Method validation included blanks, spiked samples (10, 100, 200 ng/L), and duplicates. Recoveries: PAHs: 102–117% (10 ng/L), 99–122% (100 ng/L), 101–115% (200 ng/L); SPAHs: 78–116%, 76–109%, 70–103%. Precision (intra-/inter-day): 0.4–4.0% (PAHs), 1.0–9.3% (SPAHs). Detection limits: 0.02–1.26 ng/L (PAHs), 0.13–1.98 ng/L (SPAHs).

### 2.5. Source Identification and Risk Assessment

#### 2.5.1. PCA and Isomer Ratios

Log(x + 1)-transformed and Z-score-normalized PAH/SPAH concentrations were analyzed using PCA (IBM SPSS Statistics, version 27, IBM Corp., Armonk, NY, USA). Kaiser–Meyer–Olkin (KMO > 0.6) and Bartlett’s tests (*p* < 0.05) confirmed data suitability. Principal components (eigenvalues >1) were extracted via varimax rotation [36]. Diagnostic ratios and factor regression complemented PCA for source apportionment [37].

#### 2.5.2. Risk Assessment

Risk assessment usually includes two aspects: ecological risk and health risk [38]. Ecological risks involve potential impacts on aquatic organisms, while health risks focus on potential hazards to humans after exposure through drinking water or other means [39].

Ecological risk quotient (RQ):

RQ_(NCs)_ = C_PAHs_/C_QV(NCs)_ (1)

RQ_(MPCs)_ = C_PAHs_/C_QV(MPCs)_ (2)

(3) $${RQ}_{\sum PAHs(NCs)}=\sum_{i=1}^{15}{RQ}_{\left(NCs\right)},$$

(4) $${RQ}_{\sum PAHs(MPCs)}=\sum_{i=1}^{15}{RQ}_{\left(MPCs\right)},$$

where C_PAHs_ represents the measured concentration of PAHs in surface water (ng/L); RQ_(NCs)_ and RQ_(MPCs)_ denote the minimum and maximum risk quotients, respectively; C_QV(NCs)_ and C_QV(MPCs)_ are the criteria quality values for the lowest and maximum permissible concentrations in aquatic environments (ng/L), respectively; and RQ_∑PAHs(NCs)_ and RQ_∑PAHs(MPCs)_ are the minimum and maximum risk entropy values of ∑15PAHs, respectively. The C_QV(NCs)_ and C_QV(MPCs)_ values for the 15 PAHs are listed in Table A2, while the individual PAH congeners and their ecological risk classifications are detailed in Table A3 [39,40].

The lifetime carcinogenic risk (ILCRS) model recommended by the USEPA is used to evaluate the health risks of PAHs and SPAHs in water bodies [41]. Based on the similar mechanism of toxicity of PAHs, the concentration of carcinogenic characteristic substances (c) is converted into the toxic equivalent concentration of BaP (TEQBaP), and the potential carcinogenic risks to human health in water bodies are quantitatively characterized by TEQ [18]. Among them, ingestion and skin contact are two important ways in which PAHs in water pose a threat to humans. The calculation formula and the related parameters are as follows:

(5) $$TEQ=\sum_{i=1}^{n}\left(C_{i}\times {TEF}_{i}\right),$$

(6) $$Oral\;intake:{ILCR}_{oral}=TEQ\times SF\times \frac{IR\times EF\times ED}{BW\times AT},$$

(7) $$Skin\;contact:{ILCR}_{dernal}=TEQ\times \frac{SA\times K_{p}\times ET\times EF\times ED}{BW\times AT}\times {CSF}_{dernal},$$

(8) $$\sum ILCR={ILCR}_{oral}+{ILCR}_{dernal},$$

Parameters: C, concentration (ng/L); TEF, toxic equivalency factor (Table A4); SF = 7.3; IR = 2 L/day; EF = 350 days/year; ED = 70 years; BW = 70 kg; AT = 70 years × 365 days = 25,550 days, SA = 18,000 cm^2^, Kp = 0.001 cm/h, ET = 0.58 h/day, CSF = 1.46 (mg/kg/day)^−1^. Due to the missing TEF, K_p_, and CSF values of some SPAHs, literature reports and model predictions were used for the calculation. Risk thresholds: ILCR < 10^−6^ (negligible), 10^−6^ ≤ ILCR < 10^−4^ (potential risk), ILCR ≥ 10^−4^ (high risk) [42].

## 3. Results and Discussion

### 3.1. Occurrence and Spatial Distribution of PAHs and SPAHs in Surface Water

**Figure 2:**
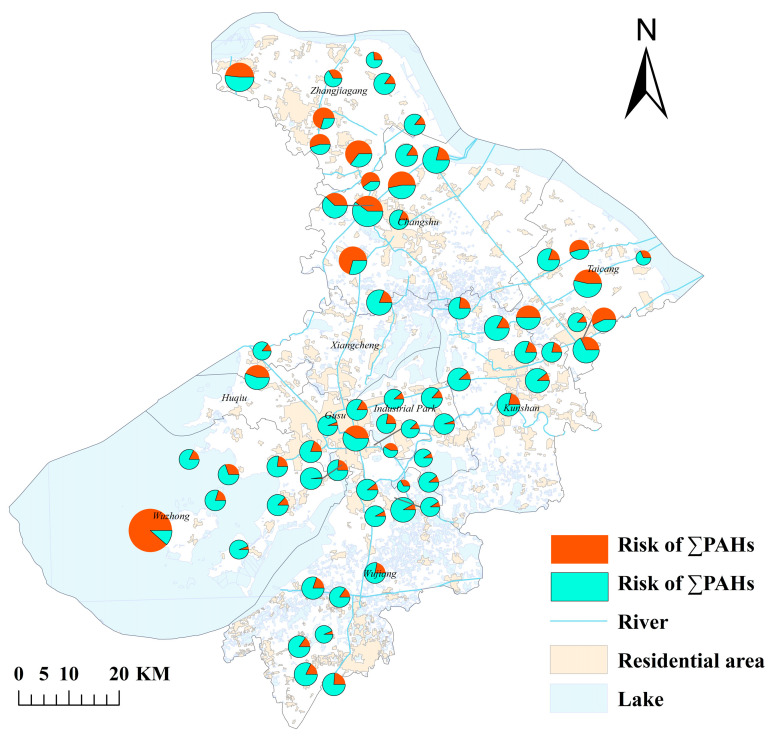
Spatial distribution of ΣPAH and ΣSPAH concentrations.

Among the collected surface water samples, 8 PAHs (NAP, ACE, ANT, PYR, BaP, PHE, FL, and INP) and 19 SPAHs were detected, while 7 PAHs (CHR, FLU, BkF, BbF, BaA, DBA, and BghiP) were below detection limits. This indicates that not all PAH congeners are present in the studied water bodies, with their distribution likely influenced by pollution sources, environmental conditions, and physicochemical properties of PAHs. The total concentration of ∑8PAHs ranged from 2.65 to 514.16 ng/L (mean: 48.05 ng/L), significantly higher than levels in the Pearl River Delta urban agglomeration (mean: 39.1 ng/L) [43] but lower than in the Bohai Bay (mean: 89.6 ng/L) [11]. The highest concentration was observed at Yali (S57, 514.16 ng/L), potentially linked to anthropogenic activities or historical pollution, while the lowest occurred at Moshe Bridge (S49, 2.65 ng/L) [33]. For ∑19SPAHs, concentrations ranged from 33.27 to 173.84 ng/L (mean: 107.43 ng/L). Elevated SPAH levels were clustered in industrial zones (e.g., Nansha Bridge, Gengjingtang Bridge), suggesting associations with traffic and combustion emissions [44] (detailed concentrations are provided in Table A5; spatial distributions are provided in Figure 2).

The geographical distribution of the sampling points (Figure 2) revealed elevated levels of NAP within ∑8PAHs in the Yali Scenic Area of Wuzhong District, Suzhou City. This may correlate with high human/vehicle traffic, water-based tourism activities, and dock operations in the area [4]. Industrial clusters (e.g., Zhangjiagang and Kunshan) exhibited significantly higher PAH and SPAH concentrations compared to other regions, with ∑19SPAHs averaging 142.6 ng/L in industrial zones, likely attributable to wastewater discharges and organic pollutant transformation [45]. Elevated NPAH levels were observed near transportation hubs (e.g., S23, Xisanhuan Lake Bridge: 31.87 ng/L; S49, Moshe Bridge: 32.98 ng/L), consistent with nitration byproducts from diesel vehicle emissions. Furthermore, it is not difficult to observe from Figure 2 that in most of the same sampling points, the phenomenon where the content of ∑SPAHs is higher than that of ∑PAHs is mainly due to the fact that PAHs can be generated and converted into SPAHs in the environment through mechanisms such as photochemical reactions and microbial interactions [46]. Their stability in the environment is usually higher than that of parent PAHs (such as NPAHs and OPAHs) [47]. In water and soil, microorganisms can degrade PAHs and generate a series of SPAHs. Some SPAHs have higher polarity and water solubility, making them easier to dissolve in water, while parent PAHs may adsorb more on suspended particles or sediments [48]. Aquatic organisms can absorb, enrich, and transform PAHs and SPAHs, and some SPAHs may have higher stability and bioaccumulation in organisms, resulting in higher concentrations of SPAHs in organisms than of parent PAHs [8]. With the increasingly strict exhaust emission control standards and the gradual popularization of new energy vehicles, the contribution of non-exhaust emissions (such as brake wear, tire wear, etc.) to traffic pollution is gradually increasing. These non-exhaust emission processes and certain industrial processes (such as coking, refining, coal combustion, etc.) may directly generate emissions of waste gas or wastewater containing SPAHs, which enter water bodies through surface runoff and other pathways [45]. These factors are intertwined and together determine the relative content of SPAHs and PAHs in water.

### 3.2. Compositional Characteristics of PAHs and SPAHs

Among the detected PAH congeners (Table A5), NAP exhibited the highest mean concentration (22.07 ng/L), followed by INP (10.48 ng/L). Notably, BaP—a potent carcinogen—showed a concentration range of ND (not detected) to 4.59 ng/L, with a detection frequency of 1.49%, indicating minimal contamination risk in the studied water bodies. Compared to global water systems, PAH concentrations in Suzhou’s surface waters were comparable to those in Shanghai rivers (46.53–221.54 ng/L) [49] and the Yellow River Delta (64.8–334.6 ng/L) [50], yet significantly lower than levels in China’s Daliao River (71.12–4255.43 ng/L) [51] and Colombia’s Cauca River (52.1–12,888.2 ng/L) [52] (Table 2). These findings suggest relatively low PAH contamination in Suzhou’s surface waters.

**Figure 3:**
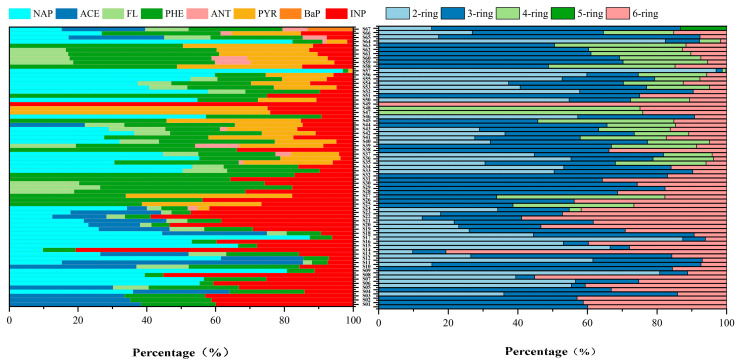
Compositions of the PAHs.

The ring distribution characteristics of PAHs in surface water were as follows: 2-ring (45.93%) > 3-ring (27.28%) > 6-ring (21.81%) > 4-ring (4.83%) > 5-ring (0.14%). Low-molecular-weight PAHs (2- and 3-ring) dominated, collectively accounting for over 70% of the total PAHs. The predominant congeners included NAP, ACE, FL, PHE, and ANT (Figure 3). Among these, NAP was the most abundant, representing 0–96.96% of the total PAHs. The prevalence of low-molecular-weight PAHs is thermodynamically governed by their higher Henry’s law constants (10^−4^–10^−5^ atm·m^3^/mol) and vapor pressures (10^−2^–10^−4^ mm Hg) [53], facilitating atmospheric deposition and water-phase partitioning. High-molecular-weight PAHs (4–6 rings) constituted 26.78% of the total PAHs. Their persistence was enhanced by hydrophobic adsorption (log Koc, 4.8–6.3) and photodegradation resistance (quantum yield Φ < 0.01 vs 0.1–0.3 for LMW PAHs) [54]. Notably, PYR (4-ring) and INP (6-ring) exhibited elevated concentrations at Xieqiao (Wang) (S20, 65.99 ng/L) and South Lake Bridge (S24, 72.43 ng/L), consistent with their particle-bound transport (98% association with PM2.5) and combustion-derived emission factors (EFPYR = 0.18–0.34 mg/kg coal) [55].

**Figure 4:**
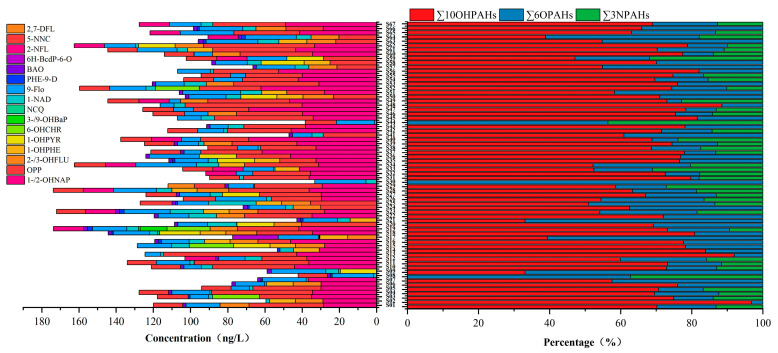
Compositions of the SPAHs.

Among the 19 SPAHs monitored, the detection rates of 1-/2-OHNAP, OPP, 1-NAD, and 9-Flo were greater than 60%, demonstrating their widespread occurrence in the studied water bodies (Figure 4). The highest mean concentration was observed for 1-/2-OHNAP (29.59 ng/L), followed by OPP (28.82 ng/L) and 9-Flo (16.69 ng/L). For SPAH subclasses: ∑10OHPAHs ranged from ND (not detected) to 128.02 ng/L (mean: 73.04 ng/L); ∑6OPAHs ranged from 1.93 to 47.07 ng/L (mean: 23.29 ng/L); and ∑3NPAHs ranged from ND to 33.08 ng/L (mean: 11.10 ng/L), with detection frequencies below 50%. OHPAHs dominated the SPAH profile, constituting 67.99% of the total SPAHs, while OPAHs and NPAHs accounted for 21.68% and 10.33%, respectively. This dominance likely stemmed from OHPAHs’ strong polarity, elevated aqueous solubility, and facilitated transformation from parent PAHs. Compared to the prior studies, the NPAH concentrations in Suzhou were lower than those in the Taihu Lake basin, industrial effluents, and municipal wastewater [56]. The OPAH levels were below those reported for Tianjin’s Haihe River [57]. Geospatial analysis revealed marginally higher SPAH concentrations near residential zones, suggesting anthropogenic influences on their distribution. However, SPAH formation is inherently linked to chemical transformations of parent PAHs, complicating direct inferences from spatial or compositional data alone [48]. Integrated multi-source analyses are thus warranted to elucidate mechanistic pathways.

### 3.3. Source Apportionment of PAHs and SPAHs

#### 3.3.1. PCA

**Figure 5:**
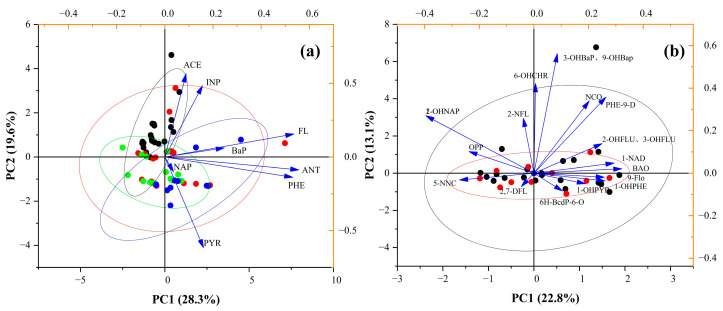
PCA of PAHs (**a**) and SPAHs (**b**) in surface water. Solid arrows represent individual PAHs (SPAHs in panel (**b**)), and dots denote sampling sites.

Source apportionment of PAHs and SPAHs was conducted through PCA combined with APCS. PCA further validated the pollution characteristics of PAHs and SPAHs (Figure 5). For PAHs, the first two principal components (PC1: 28.3%, PC2: 19.6%) cumulatively explained 47.9% of the total variance. Similarly, for SPAHs, PC1 (22.8%) and PC2 (13.1%) accounted for 35.9% of the total variance.

The six principal components (PCs) extracted from the PAH source apportionment cumulatively explained 91.84% of the total variance (Table A6). The source profiles are detailed as described below. PC1 (28.87% variance contribution): dominated by high loadings of PHE, FL, and ANT, which are established tracers of coal/coke combustion [58]; this component reflects emissions from industrial boilers and residential coal burning. PC2 (19.58%) and PC5 (9.69%): associated with BaP, ANT, and INP, indicative of high-temperature industrial processes (e.g., smelting, coking) [59]; their combined contribution (29.27%) underscores industrial dominance. PC3 (14.54%): characterized by PYR and ANT, where PYR is a diagnostic marker for diesel engine emissions, aligning with traffic-intensive sampling sites. PC4 (11.94%): primarily loaded with ACE, linked to incomplete combustion of gasoline/kerosene in regional heating or power plants. PC6 (7.22%): driven by NAP, strongly correlated with coke oven emissions and petroleum volatilization [60].

Source contributions ranked as follows: coal/coke combustion (28.87%), high-temperature industries (29.27%), traffic (14.54%), fossil fuel combustion (11.94%), and petroleum volatilization (7.22%). These results are consistent with the Jiangsu Province Industrial Emission Inventory, which reports that coal combustion contributes 26–31% of PAHs in Suzhou’s surface water, closely matching regional coking activities (Available online: http://www.suzhou.gov.cn/szsrmzf/tjgb/202303/2c908288857d4f3d01858a7e6b7a1c23.shtml (accessed on 8 May 2025)). Petroleum-related emissions align with satellite-derived estimates of oil storage terminal emissions (3.6–4.0 t/yr) in the Suzhou Industrial Park [61]. Notably, NAP, INP, and PHE collectively accounted for 81.49% of the cumulative contributions, emphasizing synergistic impacts from petroleum volatilization, industrial/traffic combustion, and diffuse combustion sources in regional pollution.

The cumulative variance explained by the eleven PCs extracted from the SPAH dataset reached 89.94% (Table A7). Their source characteristics are detailed as described below. PC1 (22.84%): dominated by 1-/2-OHNAPs, which are hydroxylated derivatives of NAP resulting from anthropogenic activities (e.g., industrial wastewater discharge) [8]. PC2 (13.11%): characterized by 3-/9-OHBaPs and NCQ, where 3-/9-OHBaPs are carcinogenic metabolites of benzo[a]pyrene, while NCQ is associated with coal tar processing and environmental oxidation of acenaphthene; this component reflects mixed contributions from industrial byproducts and biotransformation pathways. PC3 (8.67%): marked by 2-OHFLU and 1-NAD, where 2-OHFLU originates from the oxidation of high-molecular-weight PAHs during high-temperature combustion, while 1-NAD is emitted from naphthalene-containing waste in chemical production, indicating a composite source of industrial emissions and combustion byproducts [62]. PC4–PC11 (7.73%–3.75%) are mainly composed of OPP, 6H-BcdP-6-O, 1-OHPYR, 2-NFL, 6-OHCHR, 9-FLo, 5-NNC, and 2,7-DFL, respectively. Among them, OPP (PC4) is mainly produced in industrial anti-corrosion, sterilization, and other production processes, 1-OHPYR (PC6) is closely related to fossil fuel combustion, 2-NFL (PC7) is related to the diesel exhaust nitrification reaction, and 2,7-DFL (PC11) may originate from the production of fluorinated industrial products, reflecting the risk of emerging pollutants [63].

The rotated component matrices revealed complex loading patterns requiring nuanced interpretation (Table A2 and Table A3). Specifically, cross-loadings (variables loading significantly on multiple components) and negative loadings provided critical insights into source interactions. For PAHs, ANT exhibited cross-loadings across PC1 (0.527), PC2 (0.563), and PC3 (0.427), suggesting mixed contributions from coal combustion, industrial processes, and traffic emissions. Negative loadings such as PHE (−0.126 in PC4) may indicate inverse relationships between combustion sources and petroleum volatilization. In SPAHs, NCQ showed dual loadings on PC2 (0.795) and PC6 (0.350), reflecting combined industrial and biotransformation pathways. The strong negative loading of OHPHE1 (−0.666 in PC4) likely represents antagonistic effects between PAH oxidation products from different source types.

#### 3.3.2. Diagnostic Ratio

Diagnostic ratios such as Fla/(Fla + Pyr), Ant/(Ant + Phe), and InP/(InP + BghiP) have been widely applied to identify and quantify the primary sources of polycyclic aromatic hydrocarbons (PAHs).

**Figure 6:**
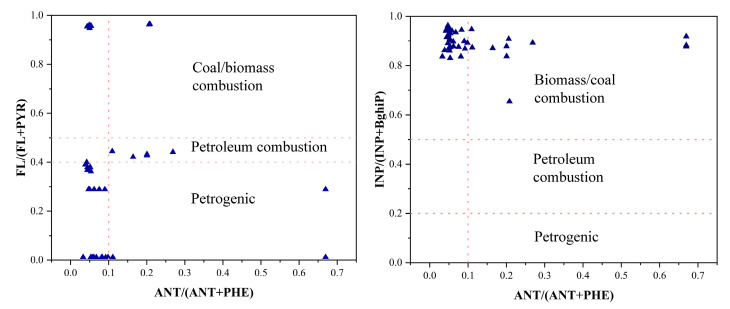
Diagnostic ratios for source identification of PAHs in the surface waters of the Suzhou.

As shown in Figure 6, the Ant/(Ant + Phe) ratios for most samples (e.g., S01–S36, S50–S63) were below 0.1, supporting a dominant petroleum origin. Similarly, Fla/(Fla + Pyr) ratios in most samples were significantly lower than 0.4, further confirming petroleum sources. However, partial samples exhibited ratios exceeding 0.5, with the maximum value reaching 0.964 (S67), unequivocally indicating high-temperature combustion of petroleum, coal, or biomass. Notably, all InP/(InP + BghiP) ratios exceeded 0.5, with over 80% of the samples surpassing 0.8, strongly implicating diesel combustion or industrial high-temperature emissions. Elevated ratios at sites near waterways—such as the Port Overpass (S06) and Chenghang Bridge (S08)—suggest ship diesel engine emissions as a key contributor. These findings align with anthropogenic activities in the Beiluo River basin, where biomass combustion for agricultural and urban-industrial energy needs releases PAHs into rivers via terrestrial runoff, atmospheric deposition, and industrial effluents [64]. Additionally, petrogenic PAHs may originate from oil spills, incomplete combustion, or extraction activities in the petroleum industry [65]. Consistent with prior studies, the primary PAH sources are linked to energy production processes (e.g., oil refining, biomass combustion) essential for industrial and agricultural development, with subsequent transport into aquatic systems through runoff, atmospheric pathways, and wastewater discharge [65,66].

### 3.4. Ecological and Health Risk Assessment

Ecological RQs were calculated for PAHs and SPAHs at various sampling points in Suzhou’s surface water. Risk levels were classified based on established PAH ecological risk thresholds. Due to the absence of C_QV(NCs)_ and C_QV(MPCs)_ for SPAHs, RQs for SPAHs were estimated using a derived formula: $RQ=\frac{C_{SPAHs}}{PENC}$ [47], which extrapolates toxicity thresholds from parent PAHs through structural-activity relationships, introducing potential uncertainties in cross-congener extrapolation accuracy.

**Figure 7:**
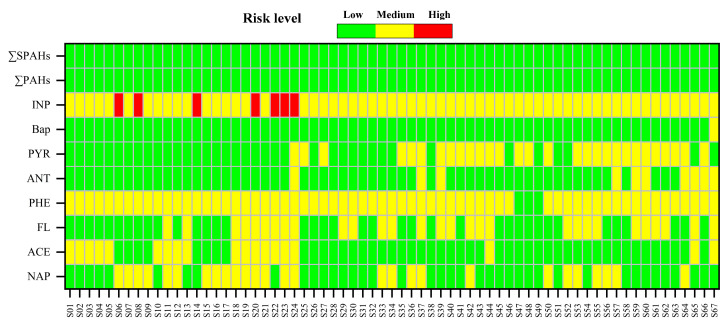
Ecological Risks of 8 PAHs, ΣPAHs, and ΣSPAHs.

The total risk quotients (∑PAHs: 5.814–213.399; ∑SPAHs: ND–1.98) indicated low cumulative ecological risks for PAHs and SPAHs in Suzhou’s surface water (Figure 7). However, moderate-to-high risks were identified for individual PAH congeners, including INP, PYR, PHE, FL, CHR, and NAP. Among these, INP exhibited the highest risk (RQ: 2.475–181.078), with seven sampling sites exceeding the high-risk threshold (RQ > 1). Although ∑SPAHs posed low cumulative risks, moderate risks were observed for specific SPAH congeners (e.g., OPP, 3-OHBaP). These hydroxylated and sulfonated derivatives demonstrated amplified bioavailability compared to parent compounds, potentially explaining their elevated per-unit risk despite lower environmental concentrations. Importantly, this study only assessed 19 SPAHs, while numerous undetected and unknown derivatives likely exist. These unmonitored compounds may amplify ecological risks, suggesting that SPAHs could pose more severe threats than parent PAHs [47,67]. Prioritized monitoring and toxicity assessments for SPAHs are thus urgently warranted in the future, particularly focusing on derivatives with reactive functional groups that may increase biological membrane permeability and metabolic activation potential.

**Figure 8:**
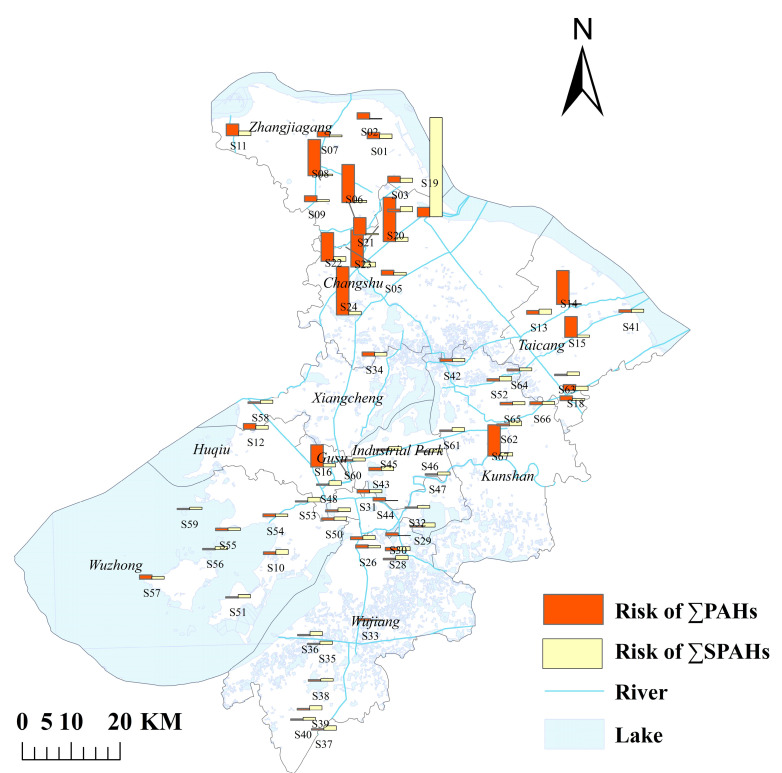
Health Risks of ΣPAHs and ΣSPAHs at 67 Sampling Sites.

Figure 8 shows the health risk analysis of PAHs and SPAHs in surface water, as can be seen from the figure we found that the PAHs risk at 67 sampling points ranged from 4.11 × 10^−8^ to 2.17 × 10^−6^, with an average of 3.47 × 10^−7^. The overall risk was not high (88.06% of low-risk areas), but there were 8 medium risk areas, possibly related to intensive industrial activities [68] or affected by shipping or surrounding emissions; When calculating the health risks of SPAHs, due to the lack of toxicological data (such as TEF and CSF) of SPAHs, this study temporarily estimated the risks based on the toxicity parameters of parent PAHs, which may lead to biased results [69]. Future experimental determination of SPAHs’ toxic effects should include microbiological toxicity tests to assess their ecological interactions and potential bioaccumulation pathways, as microbial degradation processes may alter their bioavailability and persistence in aquatic systems [67]. In the future, experimental determination of the toxic effects of SPAHs is needed to improve the evaluation. From this, it can be concluded that the risk value of SPAHs ranges from 9.63 × 10^−9^ to 4.45 × 10^−6^, with an average value of 2.09 × 10^−7^, and there is a medium risk point S19 (Gengjingtang Bridge). Due to the wide variety of SPAHs and the lack of toxicological data, as well as the fact that they are not only exposed or ingested, this study only conducted a simple risk assessment on 19 of them, which has certain limitations [9]. However, the specific risk of SPAHs showed abnormally high values at individual sites (such as S19), and their toxicity equivalent far exceeded that of the parent PAHs, indicating that substituent modification may significantly enhance the biological activity of pollutants [67]. This raises concerns about potential non-carcinogenic effects on humans, including oxidative stress, endocrine disruption, and developmental toxicity, particularly for vulnerable populations such as children and pregnant women exposed through drinking water or food chains [68,70]. This phenomenon is consistent with the toxicological enhancement trend of alkylated PAHs in recent studies [8]. The total risk value of PAHs in some high-risk areas and regions is significantly higher than the threshold, indicating that there may be hidden pollution sources around industrial intensive areas or transportation hubs, and they need to be prioritized for inclusion in the environmental supervision network. Therefore, PAHs and SPAHs have a certain potential carcinogenic risk to the human body and need to be taken seriously.

## 4. Conclusions

This study systematically investigated the occurrence, sources, and risks of PAHs and substituted PAHs (SPAHs) in surface waters of Suzhou, a rapidly urbanizing city in the Yangtze River Delta. The key findings are as follows: SPAHs surpassed parent PAHs in environmental abundance (∑19 SPAHs = 107.43 ng/L vs. ∑8 PAHs = 48.05 ng/L), with hydroxylated derivatives accounting for 67.99% of the total, indicating active environmental transformation processes. Industrial zones and transportation hubs emerged as high-risk hotspots, where 6-ring PAHs (e.g., indeno[1,2,3-cd]pyrene) and specific SPAHs (e.g., 3-OH-BaP) exhibited toxicity equivalents 2–5-fold higher than parent PAHs. Source apportionment linked 58.2% of PAHs to coal combustion and industrial emissions, demonstrating a direct correlation between contamination intensity and localized anthropogenic activities.

To address these risks, we propose implementing priority controls on industrial discharges (e.g., wastewater from chemical parks and coke production) and non-exhaust traffic emissions (e.g., tire and brake wear particles), which are critical contributors to SPAH loads. Regulatory frameworks should incorporate SPAHs, supported by expanded toxicological databases for derivatives such as hydroxylated and nitrated PAHs.

Future research directions should include multi-season sampling to assess the impact of rainfall and temperature on PAH/SPAH distribution, particularly in urban runoff systems. While this study provides a regional assessment of SPAH risks in the Yangtze River Delta, its single-season sampling design limits extrapolation to annual trends. Nevertheless, the integration of high-resolution mass spectrometry with spatial risk mapping offers a replicable framework for urban watershed management, highlighting the necessity of adapting regulatory frameworks to address substituted PAH derivatives.

## Figures and Tables

**Table 1:** Abbreviations for 15 PAHs and 19 SPAHs.

**15 a** **bbreviations for PAHs**					
Naphthalene	NAP	Chrysene	CHR	Benz[a]anthracene	BaA
Acenaphthene	ACE	Fluoranthene	FLU	Dibenz[a,h]anthracene	DBA
Anthracene	ANT	Pyrene	PYR	Benzo[a]pyrene	BaP
Phenanthrene	PHE	Benzo[k]fluoranthene	BkF	Benzo[g,h,i]perylene	BghiP
Fluorene	FL	Benzo[b]fluoranthene	BbF	Indeno[1,2,3-cd]pyrene	INP
**19 abbreviations for SPAHs**					
1-Naphthol	1-OHNAP	1-Hydroxypyrene	1-OHPYR	9-Fluorenone	9-FLo
2-Naphthol	2-OHNAP	3-Hydroxybenzo(a)pyrene	3-OHBaP	9-Phenanthraldehyde	PHE-9-D
2-Phenylphenol	OPP	9-Hydroxybenzo(a)pyrene	9-OHBaP	6H-Benzo[c,d]pyren-6-one	6H-BcdP-6-O
2-Hydroxyfluorene	2-OHFLU	1-Naphthaldehyde	1-NAD	5-Nitroacenaphthene	5-NNC
3-Hydroxyfluorene	3-OHFLU	Acenaphthenequinone	NCQ	2-Nitrofluorene	2-NFL
1-Hydroxyphenanthrene	1-OHPHE	Benzanthrone	BAO	2,7-Dinitrofluorene	2,7-DFL
6-Hydroxychrysene	6-OHCHR				

**Table 2:** Comparison of the concentrations of ΣPAHs in water with other areas.

Water (ng/L)	Location	Country	Number of PAHs	Sampling Sites	Range, ΣPAHs	Mean, ∑PAHs	Reference
	Suzhou	China	15	67	2.65–514.16	48.05	This study
	Bohai Sea	China	16	200	71.1–4260	-	[11]
	Pearl River Estuary	China	5	11	15.9–182.4	-	[43]
	Rivers in Shanghai	China	16	47	46.53–221.54	112.92	[49]
	Yellow River Delta	China	15	42	64.8–334.6	121.3	[50]
	Daliao River	China	16	27	71.12–4255.43	748.76	[51]
	Cauca	Colombia	12	8	52.1–12,888.2	2344.5	[52]

## Data Availability

Data are contained within the article. The original contributions presented in this study are included in the article. Further inquiries can be directed to the first author (corresponding author).

## Appendix A

**Table A1:** Online solid-phase extraction–liquid chromatography gradient elution program.

Time (min)	Left Pump			Right Pump			Position
	A1 (%)	B1 (%)	Flow Rate (mL/min)	A2 (%)	B2 (%)	Flow Rate (mL/min)	
−6.0	100	0	4.0	60	40	0.4	1
0.0	100	0	4.0	60	40	0.4	1
0.1	98	2	0.5	60	40	0.4	1
5.0	98	2	0.5	60	40	0.4	2
12.0	95	5	0.5	-	-	-	1
12.5	5	95	2.0	-	-	-	1
18.0	5	95	2.0	0	100	0.4	1
25.0	5	95	2.0	0	100	0.4	1
25.1	100	0	4.0	60	40	0.4	1
29.0	100	0	4.0	60	40	0.4	1

Note: −6 denotes the time required for enriching 7.5 mL of a water sample prior to data acquisition. Mobile phase: A1, ultrapure water; B1, acetonitrile; A2, ultrapure water; B2, acetonitrile.

**Table A2:** C_QV_ values of PAHs in aquatic environmental matrices.

PAHs	Water (ng/L)	
	C_QV(NCS)_	C_QV(MPCS)_
Nap	12	1200
Ace	0.7	70
Fl	0.7	70
Phe	3	300
Ant	0.7	70
Flu	3	300
Pyr	0.7	70
BaA	0.1	10
ChR	3.4	340
BbF	0.1	10
BkF	0.4	40
BaP	0.5	50
Inp	0.4	40
DBA	0.5	50
Bghip	0.3	30

**Table A3:** Ecological risk classification of individual PAHs and ΣPAHs.

Individual PAHs			∑PAHs		
Risk Level	RQ_(NCS)_	RQ_(MPCs)_	Risk Level	RQ_(NCS)_	RQ_(MPCs)_
Low risk	0	<1	No risk	0	<1
			Low risk	1–800	<1
Medium risk	≥1	<1	Medium risk 1	≥800	<1
			Medium risk 2	<800	≥1
High risk		≥1	High risk	≥800	≥1

**Table A4:** TEF and PNEC values of PAHs and SPAHs.

PAH	TEF	PNEC (ng/L)	SF	SPAH	PNEC (ng/L)
BaP	1.0	0.7	7.3	1-/2-OHNAP	100.0
				OPP	50
INP	0.1	3.0	0.73	2-/3-OHFLU	200.0
				1-OHPHE	150.0
ACE	0.01	50.0	0.073	6-OHCHR	80.0
				1-OHPYR	50.0
PHE	0.001	100.0	0.0073	3-/9-OHBaP	1.0
				9-FLo	80.0
ANT	0.01	80.0	0.073	PHE-9-D	200.0
				6H-BcdP-6-O	10.0
PYR	0.001	150.0	0.0073	5-NNC	20.0
				2-NFL	25.0
FL	0.001	600.0	0.00073	2,7-DFL	15.0
				1-NAD	300.0
NAP	0.001	1000.0	0.00073	NCQ	500.0
				BAO	50.0

**Table A5:** Concentrations and detection rates of PAHs and SPAHs in surface water.

Compound	Minimum (ng/L)	Maximum (ng/L)	Mean (ng/L)	Detection Rate (%)
NAP	ND	498.53	22.07	59.70
ACE	ND	86.95	4.41	28.36
FL	ND	7.19	1.76	47.76
PHE	ND	13.35	6.61	95.52
ANT	ND	4.98	0.33	17.91
PYR	ND	5.92	2.32	42.28
BaP	ND	4.59	0.07	1.49
INP	ND	72.43	10.48	98.51
1-/2-OHNAP	ND	68.81	29.59	76.12
OPP	ND	71.45	28.82	82.09
2-/3-OHFLU	ND	17.07	5.73	37.31
1-OHPHE	ND	15.06	3.93	28.36
1-OHPYR	ND	23.15	3.32	16.42
6-OHCHR	ND	25.33	1.42	5.97
3-/9-OHBaP	ND	14.49	0.22	1.49
NCQ	ND	1.00	0.05	7.46
1-NAD	ND	25.72	4.91	80.60
9-Flo	ND	32.64	16.69	95.52
PHE-9-D	ND	2.82	0.32	22.39
BAO	ND	2.64	0.95	49.25
6H-BcdP-6-O	ND	24.52	0.37	1.49
2-NFL	ND	16.34	3.40	20.90
5-NNC	ND	17.19	7.49	46.27
2,7-DFL	ND	14.12	0.21	1.49

**Table A6:** Total variance explained for PAHs.

Component	Initial Eigenvalues			Extraction Sums of Squared Loadings			Rotation Sums of Squared Loadings		
	Total	Variance %	Cumulative %	Total	Variance %	Cumulative %	Total	Variance %	Cumulative %
1	2.309	28.868	28.868	2.309	28.868	28.868	1.798	22.473	22.473
2	1.567	19.581	48.449	1.567	19.581	48.449	1.243	15.532	38.006
3	1.163	14.537	62.986	1.163	14.537	62.986	1.120	13.994	52.000
4	0.955	11.942	74.928	0.955	11.942	74.928	1.068	13.355	65.355
5	0.775	9.685	84.613	0.775	9.685	84.613	1.062	13.270	78.625
6	0.578	7.224	91.837	0.578	7.224	91.837	1.057	13.212	91.837
7	0.349	4.358	96.195						
8	0.304	3.805	100.000						
Rotated component matrix ^a^									
	Component								
	1		2	3		4	5		6
NAP	0.007		−0.010	0.049		0.004	0.056		0.984
ACE	0.057		0.016	−0.141		0.971	0.088		0.006
FL	0.821		0.130	0.052		0.279	0.100		−0.220
PHE	0.902		0.100	0.059		−0.126	−0.015		0.177
ANT	0.527		0.563	0.427		−0.004	0.266		0.039
PYR	0.090		−0.103	0.930		−0.152	−0.181		0.051
BaP	0.125		0.940	−0.147		0.023	−0.122		−0.022
INP	0.074		−0.067	−0.149		0.092	0.960		0.058

Rotation method: Caesar normalization maximum variance method. ^a^ The rotation converged after 6 iterations.

**Table A7:** Total variance explained for SPAHs.

Component		Initial Eigenvalues				Extraction Sums of Squared Loadings			Rotation Sums of Squared Loadings			
		Total	Variance %		Cumulative %	Total	Variance %	Cumulative %	Total	Variance %		Cumulative %
1		3.883	22.839		22.839	3.883	22.839	22.839	2.182	12.833		12.833
2		2.228	13.109		35.947	2.228	13.109	35.947	1.898	11.167		24.000
3		1.474	8.672		44.620	1.474	8.672	44.620	1.876	11.035		35.034
4		1.315	7.734		52.354	1.315	7.734	52.354	1.371	8.066		43.100
5		1.174	6.905		59.260	1.174	6.905	59.260	1.247	7.333		50.433
6		1.113	6.545		65.804	1.113	6.545	65.804	1.197	7.041		57.475
7		1.009	5.936		71.740	1.009	5.936	71.740	1.155	6.794		64.269
8		0.908	5.339		77.079	0.908	5.339	77.079	1.136	6.685		70.954
9		0.843	4.958		82.037	0.843	4.958	82.037	1.132	6.660		77.614
10		0.706	4.155		86.192	0.706	4.155	86.192	1.065	6.266		83.880
11		0.637	3.746		89.938	0.637	3.746	89.938	1.030	6.058		89.938
12		0.566	3.329		93.266							
13		0.382	2.247		95.513							
14		0.333	1.960		97.473							
15		0.268	1.574		99.047							
16		0.162	0.953		100.000							
17		2.993 × 10^−5^	0.000		100.000							
Rotated component matrix ^a^												
	Component											
	1	2	3	4	5	6	7	8	9		10	11
OHNAP1	0.945	−0.002	−0.152	0.138	−0.061	−0.093	0.069	0.066	−0.142		0.096	−0.013
OHNAP2	0.945	−0.002	−0.151	0.138	−0.061	−0.093	0.069	0.066	−0.142		.0096	−0.012
OPP	0.321	−0.092	0.156	0.847	−0.075	0.020	0.096	−0.028	−0.034		0.097	0.049
OHFLU	−0.196	0.269	0.748	0.049	0.199	0.065	0.061	−0.097	−0.211		0.011	−0.080
OHPHE1	0.032	−0.153	0.578	−0.666	0.181	0.182	0.051	0.017	0.123		−0.047	0.004
OHPYR1	−0.164	0.074	0.047	−0.044	−0.097	0.929	−0.019	0.025	0.079		−0.118	−0.018
OHCHR6	0.099	0.266	0.029	−0.045	−0.025	0.034	−0.029	0.917	−0.018		0.037	−0.011
OHBap	0.049	0.797	0.044	0.065	−0.056	−0.140	0.189	0.363	−0.023		−0.078	0.011
NCQ	−0.012	0.795	0.019	−0.045	0.226	0.350	−0.049	0.037	0.126		−0.076	−0.025
NAD1	−0.203	−0.102	0.739	0.055	−0.208	−0.034	−0.059	0.146	0.333		−0.212	−0.049
FLO9	−0.244	0.101	0.055	−0.083	0.056	0.089	0.115	−0.032	0.894		−0.013	−0.034
PHE9D	−0.121	0.506	0.435	−0.342	−0.281	−0.321	0.114	0.133	0.100		−0.089	−0.007
BAO	−0.225	0.372	0.383	−0.061	−0.329	0.041	−0.385	−0.316	0.268		−0.248	0.175
HBCDP	−0.117	0.072	0.034	−0.122	0.926	−0.079	−0.045	−0.024	0.048		−0.059	0.008
NFL2	0.095	0.114	0.036	0.044	−0.055	−0.022	0.954	−0.026	0.111		0.006	−0.020
NNC5	0.156	−0.127	−0.123	0.103	−0.059	−0.121	0.016	0.037	−0.020		0.941	0.074
DFL27	−0.018	−0.005	−0.065	0.036	0.003	−0.018	−0.029	−0.014	−0.028		0.063	0.990

Rotation method: Caesar normalization maximum variance method. ^a^ The rotation converged after 6 iterations.
